# Exogenous estradiol enhances apoptosis in regressing post-partum rat corpora lutea possibly mediated by prolactin

**DOI:** 10.1186/1477-7827-3-40

**Published:** 2005-08-30

**Authors:** Alicia A Goyeneche, Carlos M Telleria

**Affiliations:** 1 Division of Basic Biomedical Sciences, University of South Dakota School of Medicine, Vermillion, South Dakota 57069, USA

## Abstract

**Background:**

In pregnant rats, structural luteal regression takes place after parturition and is associated with cell death by apoptosis. We have recently shown that the hormonal environment is responsible for the fate of the corpora lutea (CL). Changing the levels of circulating hormones in post-partum rats, either by injecting androgen, progesterone, or by allowing dams to suckle, was coupled with a delay in the onset of apoptosis in the CL. The objectives of the present investigation were: i) to examine the effect of exogenous estradiol on apoptosis of the rat CL during post-partum luteal regression; and ii) to evaluate the post-partum luteal expression of the estrogen receptor (ER) genes.

**Methods:**

In a first experiment, rats after parturition were separated from their pups and injected daily with vehicle or estradiol benzoate for 4 days. On day 4 post-partum, animals were sacrificed, blood samples were taken to determine serum concentrations of hormones, and the ovaries were isolated to study apoptosis in situ. In a second experiment, non-lactating rats after parturition received vehicle, estradiol benzoate or estradiol benzoate plus bromoergocryptine for 4 days, and their CL were isolated and used to study apoptosis ex vivo. In a third experiment, we obtained CL from rats on day 15 of pregnancy and from non-lactating rats on day 4 post-partum, and studied the expression of the messenger RNAs (mRNAs) encoding the ERalpha and ERbeta genes.

**Results:**

Exogenous administration of estradiol benzoate induced an increase in the number of apoptotic cells within the CL on day 4 post-partum when compared with animals receiving vehicle alone. Animals treated with the estrogen had higher serum prolactin and progesterone concentrations, with no changes in serum androstenedione. Administration of bromoergocryptine blocked the increase in serum prolactin and progesterone concentrations, and DNA fragmentation induced by the estrogen treatment. ERalpha and ERbeta mRNAs were expressed in CL of day 4 post-partum animals at levels similar to those found in CL of day 15 pregnant animals.

**Conclusion:**

We have established that estradiol accelerates apoptosis in the CL during post-partum luteal regression through a mechanism that possibly involves the secretion of pituitary prolactin. We have also shown that the post-partum rat CL express ERalpha and ERbeta mRNAs suggesting that they can be targeted by estrogen.

## Background

The regression of corpora lutea (CL) is a process that involves two stages. During the first stage (functional regression), production of progesterone is discontinued. In the second stage (structural regression), the CL undergo involution manifested by a decrease in weight and size that is associated with programmed death of the luteal cells [1-6]. In the rat CL, programmed cell death follows a pattern of death by apoptosis characterized by initial condensation of the nuclear chromatin followed or accompanied by nucleosomal fragmentation of DNA and formation of apoptotic bodies, which eventually are eliminated by phagocytosis [7,8].

In the regressing CL of pregnancy, apoptosis is a lengthy process that occurs over the course of many days from the initial decrease in the progesterone producing capacity of the glands, to the decrease in their sizes. As a consequence, the structural changes of the CL undergoing regression are usually studied after parturition [8-10]. The rat ovulates within 24–36 h following parturition [11]. Therefore, when studying luteal regression after parturition, two populations of CL can be analyzed simultaneously, the CL of previous pregnancy and the CL formed after post-partum ovulation [8,12]. We have shown previously that the two populations of CL found within the post-partum ovary have similar rate of apoptosis despite their difference in age [10].

The regression of the CL in the rat ovary after parturition is hormonally regulated. We demonstrated that luteal apoptosis in this species can be accelerated by the administration of either the antigestagen RU486 or prostaglandin F_2α _[7], both of which induce large declines in the capacity of the CL to produce progesterone. Conversely, we and others have shown that the onset of apoptosis in the post-partum CL can be delayed by administration of androstenedione [9], progesterone [10], or by allowing the dams to suckle [8,12].

During pregnancy in rats, circulating concentration of estradiol increases on day 3, after which remains very low until day 15–16 when it starts to increase progressively towards parturition [13,14]. Moreover, the pregnant rat CL express estrogen receptors (ERs) alpha (ERalpha) and beta (ERbeta) under the regulation of prolactin and placental lactogens [15], and respond to estradiol, which stimulates steroidogenesis [16,17], mediates luteal cell hypertrophy by increasing protein biosynthesis [18], and synergizes the luteotropic effect of prolactin [19]. Whether estradiol regulates luteal function during luteal regression, and whether ERs are expressed in the regressing CL of the post-partum rat, is presently unknown. Therefore, in the present investigation we studied the effect of exogenous estradiol benzoate on apoptosis of regressing CL post-partum and whether these CL express ERalpha and ERbeta mRNAs.

## Materials and methods

### Animals

Pregnant (day 1 = sperm positive) Sprague-Dawley rats were obtained from Harlan Labs (Indianapolis, IN, USA). They were housed under controlled conditions of light (lights on 05:00–17:00 h) and temperature (21–23°C) with free access to standard rat chow and water. Animals were killed by decapitation and handled in conformance with the Guide for the Care and Use of Laboratory Animals, National Academy of Sciences, USA, 1996. The experimental protocol was approved by the University of South Dakota Animal Care and Use Committee.

### Experimental procedure

To determine the effect of estrogen on luteal apoptosis, two groups of post-partum rats were used, each composed of 6 to 8 animals. The pups were removed immediately after parturition, and the rats were injected daily with estradiol benzoate (5 μg/rat s.c.) or vehicle (sunflower seed oil) at 10:00 h. Animals were killed by decapitation at 13:00 h on day 4 post-partum. Trunk blood was obtained to determine hormone concentrations. The ovaries were removed and fixed for 1 h at room temperature in 4% paraformaldehyde, dehydrated in ethanol series, cleared in xylene, and embedded in paraffin for routine hematoxylin and eosin (H&E) staining. Luteal regression was evaluated separately in the two generations of CL (i.e., CL of the previous pregnancy and new CL formed after ovulation post-partum) by studying the number of nuclei undergoing apoptosis. Under the light microscope, the old CL of pregnancy have organized cell distribution and closed capillaries, whereas the newly formed CL have a less organized cell distribution and open capillaries. Two additional groups of post-partum rats, each composed of 6 to 8 animals, were treated and sacrificed as described above, and the CL were isolated from the ovaries under a stereoscopic microscope and weighed. The CL of the previous pregnancy were recognized from the newly formed CL as they were larger and less vascularized.

In a second experiment three groups of rats, each composed of 9 to 10 animals, were treated daily after parturition with vehicle, estradiol benzoate (5 μg/rat s.c.) or estradiol benzoate plus bromoergocryptine (0.5 mg/rat s.c.) at 10:00 h. Animals were killed by decapitation at 13:00 h on day 4 post-partum. Trunk blood was obtained to determine hormone concentrations. The ovaries were removed and the CL of previous pregnancy were isolated under a stereoscopic microscope, pooled and used for ex vivo incubation. The isolation of CL of the previous pregnancy was performed as previously reported [10]. From the analysis of the first experiment we concluded that the incidence of apoptosis in both CL subtypes evaluated in situ was similar. Therefore, for this experiment, only CL of the previous pregnancy were used, since they are larger and easier to isolate from the ovarian stroma than those formed after post-partum ovulation.

In a third experiment, 3 rats per group were sacrificed at 13:00 h on day 15 of pregnancy and on day 4 post-partum. In the latter group of rats, the pups were removed immediately following parturition. The CL were isolated from the ovaries under a stereoscopic microscope, frozen in liquid nitrogen, and stored at -80°C until processed for RNA isolation.

### Counting of apoptotic cells

Apoptotic cells were counted in H&E stained tissue sections on the basis of morphological criteria using an optical microscope as previously described [8]. Briefly, only cells with advanced signs of apoptosis (i.e., containing multiple nuclear fragments) were counted. A microscope with a 100 × objective was used and all fields were analyzed in each CL for the presence of fragmented nuclei. All the CL in each section were studied, and an average number of apoptotic nuclei per high power field was obtained. The expression of apoptosis per field rather than per CL more accurately reflects the dynamics of the apoptotic process within each CL in a size-independent manner at any given time after parturition. This morphometric method for the identification of apoptotic cells was previously validated in the CL by in situ 3' end labeling [7].

### Incubation of CL

The CL (four to six per well in a 24-well tissue-culture plate) were incubated in serum-free medium (McCoy 5A: Ham F12, 1:1, v/v; Sigma Chemical Co., St. Louis, MO, USA) containing 25 mM Hepes, 200 IU/ml penicillin G, 200 μg/ml streptomycin, and 0.5 μg/ml of amphotericin B at 37°C for various periods of time in an atmosphere of 95% air/5% CO_2_. After incubation, the CL were immediately frozen in liquid nitrogen and stored at -80°C until DNA isolation.

### DNA fragmentation

The internucleosomal cleavage of the DNA was analyzed as follows: the CL of previous pregnancy were isolated on day 4 post-partum and were digested overnight at 50°C in a buffer composed of 100 mM NaCl, 10 mM Tris HCl (pH 8.0), 25 mM EDTA (pH 8.0), 0.5% SDS, and 0.1 mg/ml proteinase K (Life Technologies, Rockville, MD, USA). The genomic DNA was extracted from the digested tissues with phenol/chloroform/isoamyl alcohol (25:24:1, v/v/v), precipitated, and digested for 1 h at 37°C in 1 μg/ml of ribonuclease from bovine pancreas (deoxyribonuclease-free; Roche, Indianapolis, IN, USA). After extraction and precipitation, an equal amount of DNA for each sample (1 μg) was separated by electrophoresis on a 2% agarose gel, impregnated with SYBR Gold nucleic acid gel stain (Molecular Probes, Eugene, OR, USA), examined using an ultraviolet transilluminator, and photographed with the Amersham Typhoon fluorescence imaging system (Amersham Biosciences Corp., Piscataway, NJ, USA). A 100-base pair (bp) DNA ladder (Promega, Madison, WI, USA) was used for determining the size of the DNA fragments. The UN-SCAN-IT gel software (Silk Scientific, Inc., Orem, UT, USA) was used to semiquantitate the fragmented DNA. The densitometry of the DNA fragments that appeared below 2070 bp was recorded. This measurement was normalized against the density of the total genomic DNA of the sample to correct for DNA loading. The density of the total genomic DNA of each sample was calculated as the sum of the density of the DNA fragments below 2070 bp plus the density of the large DNA band found above the 2070 bp marker. In addition, to allow comparisons between different gels, the normalized densitometric values for each of the incubation times were divided by the normalized densitometric values at time zero of incubation. The samples of DNA used for time zero of incubation were obtained from CL that were isolated for ex vivo incubation but were frozen without being incubated.

### Hormone assays

Estradiol concentrations were measured by radioimmunoassay (RIA) using a commercially obtained kit (Diagnostics Systems Laboratories, Webster, TX, USA). The sensitivity was 2.2 pg/ml, and the inter- and intra-assay coefficients of variation were 7.5% and 9.3% respectively. Androstenedione was assayed using a RIA previously described [9]. The sensitivity of the assay was 6 pg/tube and the intra- and inter-assay coefficients of variation were 4.4% and 16.7% respectively. Progesterone was assayed by enzyme immunoassay (EIA) using a commercially obtained kit (Cayman Chemical, Ann Arbor, MI, USA). The sensitivity of the assay was 10 pg/ml, and the inter- and intra-assay coefficients of variation were 5% and 6% respectively. Rat prolactin was assayed by EIA using a commercial kit (SPIbio, Massy Cedex, France). The sensitivity of the assay was 0.5 ng/ml and the intra- and inter-assay coefficients of variation were 8.1% and 14% respectively.

### RNA isolation and reverse transcription polymerase chain reaction

The CL from rats on day 15 of pregnancy or on day 4 post-partum were obtained and total RNA was extracted using TRIzol (Invitrogen, Carlsbad, CA, USA) per manufacturer's instructions. Reverse transcription (RT) reaction and polymerase chain reaction (PCR) were done using a SuperScript One-Step RT-PCR system with Platinum Taq (Invitrogen). PCR was carried out in a GeneAmp PCR 2700 thermal cycler (Applied Biosystems, Foster City, CA, USA) for 25 cycles using 61°C as the annealing temperature. The conditions were such that the amplification of the products was in the exponential phase, and the assay was linear with respect to the amount of input RNA. Oligonucleotide primer pairs were based on the sequence of the rat ERalpha gene (5'-AATTCTGACAATCGACGCCAG-3') and (5'-GTGCTTCAACATTCTCCCTCCTC-3'), the rat ERbeta gene (5'-AAAGCCAAGAGAAACGGTGGGCAT-3') and (5'-GCCAATCATGTGCACCAGTTCCTT-3'), and the rat ribosomal protein L19 (used as a housekeeping gene) (5'-CTGAAGGTCAAAGGGAATGTG-3') and (5'-GGACAGAGTCTTGATATCTC-3'), as previously described [15,20]. The predicted sizes of the PCR-amplified products were 344 (ERalpha), 204 (ERbeta) and 194 (L19). The PCR products were run on a 2.5% agarose gel, stained with SYBR Gold nucleic acid gel stain (Molecular Probes), and photographed with the Amersham Typhoon imaging system (Amersham). A 100 bp DNA ladder (Promega) was used for determining the size of the PCR products.

### Statistics

Comparisons between means of two groups were carried out using Student *t*-tests. For multiple comparisons, one-way analysis of variance followed by Tukey's or Dunnet's multiple comparison test was used. A difference was considered to be statistically significant at *P *< 0.05.

## Results

### Potentiation of apoptosis by estradiol benzoate in the post-partum CL

**Figure 1:**
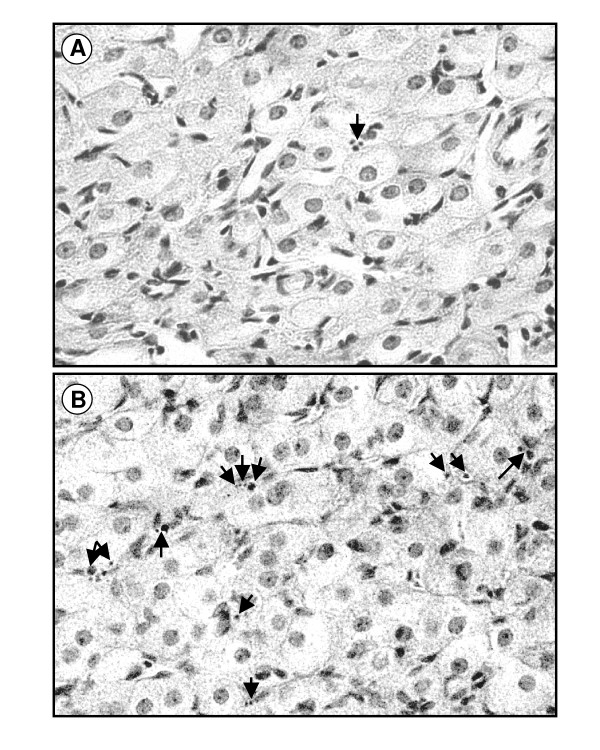
**Micrographs of CL obtained from rats killed on day 4 post-partum and stained with H&E**. Panel (A) shows the structure of a an old CL of pregnancy obtained from an animal treated with vehicle, whereas panel (B) displays the structure of an old CL of pregnancy obtained from a rat treated with estradiol benzoate. Similar results were observed in newly formed CL after post-partum ovulation obtained from animals receiving vehicle or estradiol benzoate. The arrows indicate nuclei undergoing apoptosis displaying different features of chromatin condensation. Magnification × 400.

**Figure 2:**
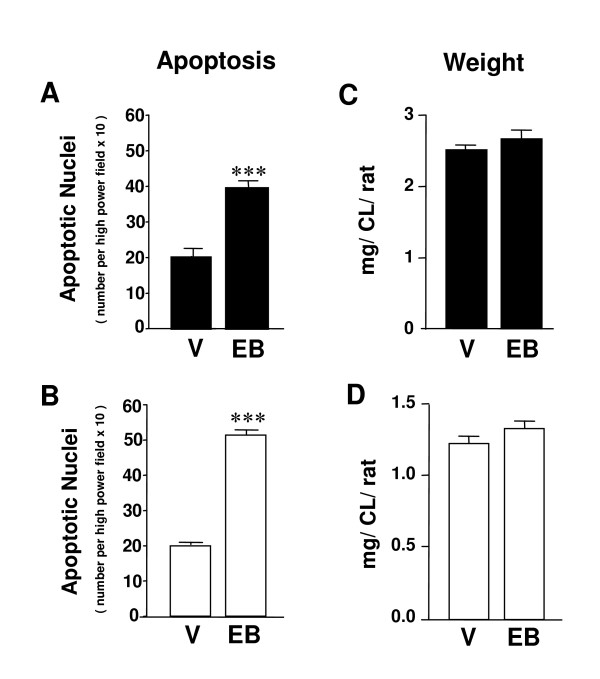
**Effect of treatment with estradiol benzoate on CL apoptosis and weight in the rat ovary after parturition**. Ovaries were obtained at 13:00 hon day 4 post-partum from animals that had received vehicle (V) [non-lactating animals treated daily with sunflower seed oil at 10:00 h (n = 6–8)], or estradiol benzoate (EB) [non-lactating animals treated daily with estradiol benzoate (5 μg/rat s.c.) at 1000 h (n = 6–8)]. The ovaries were processed for routine H&E staining, and the number of apoptotic figures was counted under a light microscope (A and B). The average weight of the CL was recorded in two other groups of 6 to 8 animals (C and D). Filled bars represent old CL of gestation, whereas open bars represent newly formed CL after ovulation post-partum. *** p < 0.001 compared with vehicle (Student *t*-test).

To study the effect of estradiol on apoptosis during luteal regression, we used rats whose pups were removed immediately after delivery to prevent the initiation of lactation. Under these conditions, the animals undergo extensive luteal regression in both generations of CL (i.e., old CL of previous pregnancy and newly formed CL after post-partum ovulation) by day 3–4 post-partum [10,21]. Figure 1 shows representative images of H&E stained sections from CL of non-lactating animals treated with vehicle or estradiol benzoate and sacrificed on day 4 after parturition. The CL of vehicle-treated controls displayed few apoptotic nuclei per microscope field (Figure 1A), whereas treatment with estradiol benzoate markedly enhanced the number of apoptotic figures (Figure 1B). The images depicted in this figure correspond to an old CL of pregnancy, but similar images were observed in newly formed CL after post-partum ovulation (data not shown). Figures 2A and 2B show the quantification of apoptotic nuclei found within both generations of CL in vehicle-treated rats and in rats receiving estradiol benzoate. The average weight of the CL seen in control animals on day 4 post-partum was not affected by treatment with the estrogen (Figures 2C and 2D).

### Circulating concentrations of 17beta-estradiol, androstenedione, progesterone and prolactin in animals treated with estradiol benzoate after parturition

**Figure 3:**
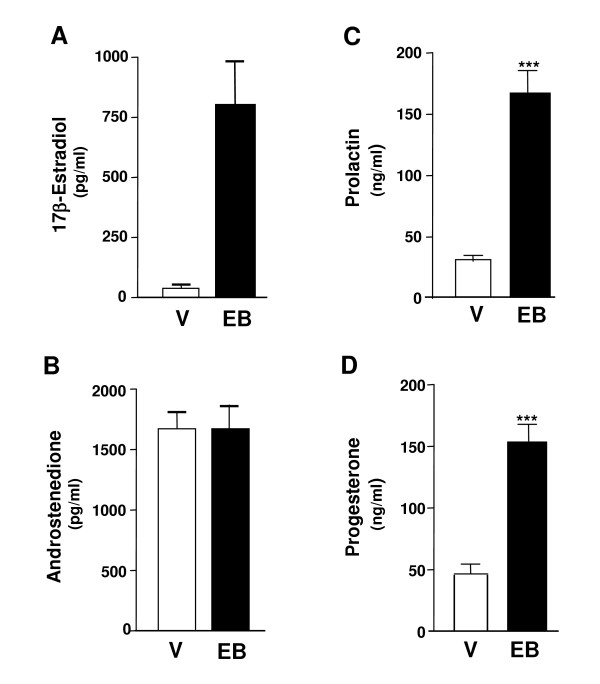
**Serum hormone concentrations in post-partum non-lactating animals treated with vehicle or estradiol benzoate**. 17beta-estradiol (A), androstenedione (B), prolactin (C) and progesterone (D) concentrations in serum of non-lactating animals treated daily at 10:00 h with either vehicle (V) or estradiol benzoate (EB; 5 μg/rat s.c.), and killed at 13:00 h on day 4 post-partum. *** p < 0.001 compared with vehicle-treated animals (Student *t*-test).

Animals that were treated with estradiol benzoate, as expected, displayed elevated circulating levels of 17beta-estradiol (Figure 3A). Androstenedione, shown to be capable of interfering with luteal regression in post-partum rat CL [9], did not change with estrogen treatment (Figure 3B). Prolactin, one of the main hormones involved in CL function in rats, had a significant increase in its circulating concentration after treatment with estradiol benzoate (Figure 3C), in parallel with a significant increase in circulating levels of progesterone (Figure 3D).

### Bromoergocryptine abrogates estradiol benzoate-induced increase in serum prolactin and progesterone concentrations in post-partum rats

Because it is known that in pregnant rats prolactin stimulates the production of progesterone by the CL [22], we evaluated whether the enhanced circulating levels of progesterone in response to treatment with estradiol benzoate were the consequence of a direct effect of the estrogen on the ovary, or instead, an indirect effect mediated by prolactin secreted in response to estradiol. To answer this question, we injected post-partum animals with estradiol benzoate plus bromoergocryptine in order to block the production of pituitary prolactin induced by the estrogen. The serum concentrations of prolactin and progesterone, which were increased by administration of estradiol benzoate, were abrogated by bromoergrocryptine, reaching control levels (Table 1). These results suggest that the increase in the production of progesterone in estrogen-treated animals is most likely a consequence of an effect of prolactin rather than a direct effect of estradiol on the ovary.

**Table 1:** Effect of bromoergrocryptine (BEC) on serum concentrations of progesterone and prolactin in post-partum non-lactating rats treated with estradiol benzoate (EB).

	**Vehicle**	**EB**	**EB + BEC**
Progesterone (ng/ml)	45.9 ± 7.2 (10)	154 ± 13.3 (10) ***	44.6 ± 6.7 (9)
Prolactin (ng/ml)	30.2 ± 4.2 (10)	166 ± 19.2 (10) ***	25.5 ± 6.5 (9)

Values are mean ± SEM for the number of animals in parentheses. *** p < 0.001 compared with groups receiving vehicle or EB + BEC (one-way analysis of variance followed by the Tukey multiple comparison test).

### Bromoergocryptine abrogates estradiol benzoate enhancement of ex vivo-induced luteal DNA fragmentation

**Figure 4:**
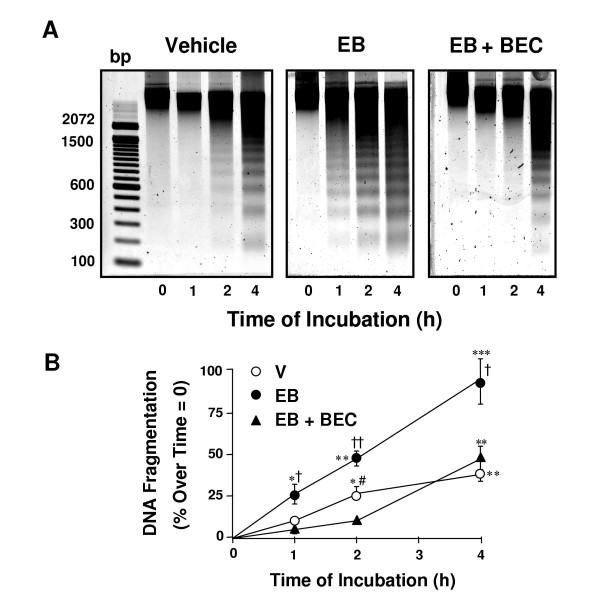
**Effect of estradiol benzoate or estradiol benzoate plus bromoergocryptine on DNA fragmentation in CL incubated in serum-free conditions**. At the end of the incubation, genomic DNA was extracted from vehicle-treated, estradiol benzoate (EB)-treated or EB plus bromoergrocriptine (BEC)-treated animals and run on a gel (Panel A). Densitometry of the DNA fragments studied from three different experiments with similar outcome is also shown (Panel B). * p < 0.05, ** p < 0.01 and *** p < 0.001 compared with values at time zero (one way analysis of variance followed by the Dunnett multiple-comparison test). † *P *< 0.05 and †† *P *< 0.01 compared with the corresponding time across treatments (one way analysis of variance followed by the Tukey multiple comparison test). bp, Base pairs. Only CL of previous pregnancy were evaluated.

To further study the effect of estradiol on apoptotic cell death in the rat CL, we used a previously defined ex vivo approach in which CL incubated in serum-free conditions accumulate large number of cells in different stages of apoptosis. This approach is sensitive to study hormonal regulation of apoptosis in the CL [8-10,23]. It takes 2 to 4 h of organ culture to observe DNA fragmentation [8], whereas the length of this period depends upon the hormonal environment to which the gland had been exposed in vivo. In the current work, old CL obtained from non-lactating rats on day 4 post-partum and incubated in serum-free conditions displayed DNA fragmentation in a time-dependent manner (Figure 4A, left panel, and 4B). However, in non-lactating rats that had received a daily injection of estradiol benzoate from days 1 to 4 post-partum, the ex vivo-induced luteal DNA fragmentation was markedly accelerated and more abundant (Figure 4A, middle panel, and 4B). When the animals were treated with estradiol benzoate in conjunction with bromoergocryptine, the temporal pattern of DNA fragmentation ex vivo, as well as the abundance of fragmented DNA, was comparable to that of vehicle-treated animals (Figure 4A, right panel, and 4B). These data indicate that the blockage of estradiol-induced prolactin secretion prevents fragmentation of DNA induced by the estrogen, and suggest that the pro-apoptotic signal triggered by estrogen on the post-partum CL is most likely the consequence of the action of prolactin.

### Expression of ER in the post-partum rat CL

**Figure 5:**
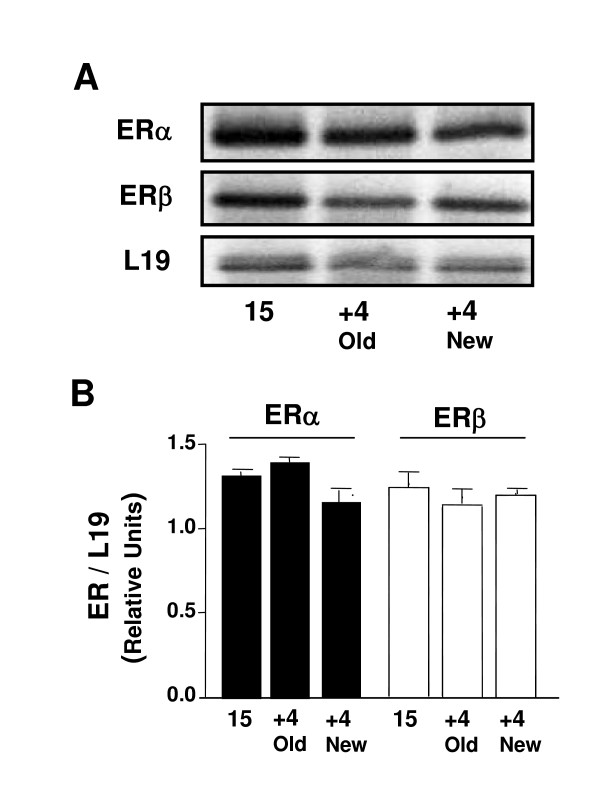
**Expression of ERalpha and ERbeta mRNA in the two populations of CL found in the post-partum ovaries**. Old indicates CL from previous pregnancy. New indicates newly formed CL after post-partum ovulation. Total RNA was isolated from old or new CL on day 4 post-partum, and from CL obtained from animals on day 15 of pregnancy, and was analyzed by RT-PCR. In panel (A) a representative gel is shown. Results were quantified by densitometry and corrected using L19. Normalized mRNA levels are graphically represented in panel (B) as the mean ± SEM (n = 3).

To determine whether ERalpha and ERbeta genes are expressed in the rat CL post-partum, we performed semiquantitative RT-PCR to specifically detect the ERalpha and ERbeta mRNA transcripts. As a positive control for ER mRNA expression we used CL obtained from day 15 pregnant rats, which were previously described as expressing both ER mRNA transcripts [15]. Figure 5A shows that ERalpha and ERbeta mRNA transcripts are highly expressed 4 days after parturition in both types of CL found within the ovaries. Semiquantitative analysis (Figure 5B) shows no difference between the abundance of each ER transcript on the different days analyzed as well as when comparing new and old types of CL on day 4 post-partum. These data also indicate that the post-partum CL can be direct targets of estrogen action.

## Discussion

The pro- or anti-apoptotic effects of estrogens are controversial. For example, estradiol enhances neuronal survival [24,25], but sensitizes anterior pituitary gland to apoptosis [26]. We have shown that estradiol enhances apoptosis in regressing CL of rats. In rabbits, however, estradiol protects the CL from apoptosis [27]. Thus, the effect of estradiol on apoptosis appears to be cell and species dependent.

A role for estradiol in the induction of apoptosis has been suggested for the CL of primates. Prior to the induction of apoptosis the human CL express high levels of 17beta-hydroxysteroid dehydrogenase type I, the enzyme that converts estrone to estradiol [28], suggesting a role for locally produced estradiol in triggering luteal regression. An increase in the sensitivity to estradiol has also been proposed in the CL of monkeys coincident with luteal regression [29]. Further, in a recent review on apoptosis in the human ovary, a role for estradiol has been suggested in triggering luteal regression [30]. Our results in regressing rat CL support a similar effect of estradiol to that shown in humans, stimulating luteal regression through the promotion of luteal apoptosis, and suggest that the elevated estrogenic environment to which the CL of rats are exposed at the time of parturition [13,14] may play a role in the post-partum facet of luteal regression.

In our studies, the pro-apoptotic effect of estradiol appears to rely on the presence of pituitary prolactin. The stimulation of prolactin secretion by estradiol in rats has been demonstrated in several in vivo settings. For example, estradiol implants made in the arquate nucleus provoke a sustained release of prolactin [31], whereas systemic administration of estradiol benzoate in ovariectomized rats stimulates prolactin secretion by a mechanism that involves the opioid system [32]. Furthermore, estradiol can also stimulate prolactin synthesis and release by directly targeting the lactotrophes [33].

One interesting finding in our study is that exogenous estradiol increased apoptosis in the CL while at the same time increased serum progesterone levels. An interaction between estrogen, progesterone and prolactin has been shown during the regression of the CL of the rat estrous cycle [34]. These authors demonstrated that antiestrogens decrease the detrimental effect of prolactin on the CL, whereas progesterone favors the action of prolactin, suggesting that an estrogenic and progestational hormonal environment favors apoptosis induced by prolactin. Our results are in keeping with this because the increased apoptosis induced by estradiol in the post-partum rat CL also occurred in the presence of elevated concentrations of progesterone and prolactin.

The effect of estrogen on progesterone production in rats varies with the experimental approach. For example, in hypophysectomized and hysterectomized rats estradiol stimulates the production of progesterone [35]. In contrast, in pseudopregnant rats, treatment with estradiol benzoate decreases plasma levels of progesterone [36], and, in pregnant rats, estradiol given from days 7 to 14 of pregnancy reduces the levels of circulating progesterone measured on day 15 of pregnancy [37]. In our study using intact rats after parturition estrogen treatment increased progesterone production and such effect seems mediated by pituitary prolactin because it could be abrogated by bromoergocryptine.

Prolactin has different effects on the CL depending on the prevailing steroid concentrations. For example during lactation, prolactin prevents apoptosis in the CL [8,12]. This effect of prolactin occurs in the presence of high circulating levels of progesterone [8], at least until day 9 of lactation [12]. In this physiological situation, estradiol levels are low and beginning to increase towards the 8^th ^to 10^th ^day of lactation [38]. Conversely, our studies in non-lactating post-partum rats receiving estradiol suggest that prolactin stimulates rather than prevents luteal apoptosis, and that this effect takes place in the presence of high progesterone. Together these studies suggest that prolactin may stimulate progesterone production, but at the same time may either trigger or prevent luteal apoptosis depending upon the estrogenic background. Whereas a high estrogenic background may favor a pro-apoptotic action of prolactin (e.g. as in the cyclic rat CL), a low estrogenic background may favor an anti-apoptotic effect of the hormone (e.g. as in the CL of lactation). Further studies need to be done to prove or disprove this hypothesis, but answering this question may resolve the unclear issue that prolactin appears to have a dual effect in the rat CL, being both a survival factor [8,20,22,39] and a pro-apoptotic factor [3,40-46]. It is feasible that opposite actions of prolactin in the CL, driven by the estrogenic environment, are mediated through different signal transduction pathways activated by the lactogenic hormone upon binding to its luteal receptor subtypes [20,47], or by differential activation and turnover of transcription factors [48].

A direct effect of prolactin on the regressing CL after parturition is supported by the presence of receptors for prolactin in both old CL of pregnancy and newly formed CL after ovulation post-partum [8]. Whereas the expression of prolactin receptors in estrogen-treated animals was not measured in the present study, we can anticipate their presence. This is because prolactin has been shown to up-regulate its own receptors in the CL [20]; thus, it is unlikely that in an environment high in prolactin as observed in estrogen-treated rats, those receptors would have been reduced.

In the present study, we have shown that the ER genes are expressed in post-partum CL making them potential direct targets for the action of estradiol. As we only studied the expression of the ER mRNAs, we cannot conclusively indicate that the ER proteins follow similar expression pattern. Yet, most probably the latter assumption is true because a correlation was shown between expression of ER message and protein in the pregnant rat CL [15]. The rat CL express ERalpha and ERbeta mRNAs and ER immunoreactive proteins along pregnancy, with a decline in expression occurring at parturition [15]. Because the post-partum CL express ER transcripts at levels similar to the levels measured at mid-pregnancy, it can be suggested that the down-regulation of ER expression in the rat CL is limited to parturition.

Previously, we demonstrated that the main circulating androgen in female rats, androstenedione, protects the post-partum CL from undergoing apoptosis [9]. In the present report we show that estradiol enhances apoptosis in the same CL types. Taken together, the results of these two studies suggest that androgens and estrogens oppose each other's action on luteal apoptosis. This opposite effect of androgens and estrogens also occurs in granulosa cells, with however, a different pattern. Rat granulosa cells maintained in culture respond to testosterone with an increase in apoptosis, and to estradiol with a decrease in apoptosis [49]. Whereas an increase in the androgen to estrogen ratio in the follicular fluid favors apoptosis of granulosa cells and overall follicular atresia [50,51], a similar change in ratio within the CL may protect the gland from regression.

## Conclusion

We have shown that estradiol increases apoptosis in the rat CL during post-partum luteal regression and presented evidence suggesting that pituitary prolactin is the mediator of such effect. We also demonstrated the expression of ERalpha and ERbeta mRNA transcripts in the two populations of CL found in this species after parturition (i.e. the newly formed CL after post-partum ovulation and the old CL of pregnancy). Moreover, the data suggest that the estrogenic environment might be a key factor driving a detrimental effect of prolactin in the post-partum CL.

## Authors' contributions

AAG participated in the design of the study and carried out most of the experiments. CMT conceived the study, measured hormone concentrations, carried out some of the experiments, and wrote the manuscript. Both authors read and approved the final manuscript.
